# The Contribution of Immune Infiltrates to Ototoxicity and Cochlear Hair Cell Loss

**DOI:** 10.3389/fncel.2017.00106

**Published:** 2017-04-12

**Authors:** Megan B. Wood, Jian Zuo

**Affiliations:** Department of Developmental Neurobiology, St. Jude Children’s Research HospitalMemphis, TN, USA

**Keywords:** ototoxicity, CX3CR1+ macrophage, TLR4, noise-induced hearing loss, sterile inflammation

## Abstract

Cells of the immune system have been shown to infiltrate the cochlea after acoustic trauma or ototoxic drug treatment; however, the contribution of the immune system to hair cell loss in the inner ear is incompletely understood. Most studies have concentrated on the immediate innate response to hair cell damage using CD45 as a broad marker for all immune cells. More recent studies have used RNA sequencing, GeneChip arrays and quantitative PCR to analyze gene expression in the entire cochlea after auditory trauma, leading to a better understanding of the chemokines and cytokines that attract immune cells to the cochlea. Immune suppression by blocking cytokines or immune receptors has been proven to suppress hair cell damage. However, it is now understood that not all immune cells are detrimental to the cochlea. CX3CR1+ resident macrophages protect hair cells from damage mediated by infiltrating immune cells. Systemically, the immune response is associated with both protection and pathology, and it has been implicated in the regeneration of certain tissues after injury. This review focuses on the studies of immune cells in various models of hearing loss and highlights the steps that can be taken to elucidate the connection between the immune response and hearing loss. The interplay between the immune system and tissues that were previously thought to be immune privileged, such as the cochlea, is an emerging research field, to which additional studies of the immune component of the cochlear response to injury will make an important contribution.

## Introduction

In the cochlea, the loss of sensory hair cells in the organ of Corti or the connecting spiral ganglion neurons due to exposure to ototoxic drugs or excessive noise results in hearing loss. There is increasing evidence that the loss of one or both of these cell types is exacerbated by inflammation of the cochlea. The direct action of infiltrating immune cell types and their cytokines, as well as reactive oxygen species (ROS) and cytokines generated by resident cochlear cells, leads to irreparable damage to hair cells and neurons (Bánfi et al., 2004; Lang et al., 2016). Understanding the cell types and cellular products that lead to this cell death will provide valuable targets for combatting sensorineural hearing loss. Although Fredelius and Rask-Andersen (1990) first reported the infiltration of immune cells into the noise-damaged cochlea nearly 30 years ago, the phenomenon has attracted renewed interest in the last 10–15 years. The following review discusses the recent advances in our understanding of the role of the cells of the immune system in hearing loss due to noise and ototoxic drug treatment. It is hoped that our examination of the existing literature will serve as a basis for developing new ideas in this exciting area of research that may ultimately lead to new ways of preventing or treating hearing loss resulting from the aforementioned causes.

## Basic Pathways in Sterile Inflammation

Inflammation in the ear caused by exposure to ototoxic drugs or excessive noise is unique in that the resulting immune response at the epithelial surface is not a response to a pathogen. Accordingly, it is termed sterile inflammation (Ma et al., 2000; Rock et al., 2010). In the past, it was thought that the immune system was unable to infiltrate into several sites of the body. Such “immune privileged” sites included the brain, the inner ear, the eye and the joint capsules among others. However, the multiple observations of immune responses in these sites have fundamentally changed our understanding, such that the immune system is now thought to be able to respond in all tissues, albeit with varying degrees of efficiency (Galea et al., 2007; Taylor, 2016). Furthermore, many of the tissues formerly considered to be “immune-privileged” have their own specific resident immune cell populations, with the microglia of the brain being the best characterized in this regard (Immig et al., 2015). When damage occurs in these tissues in the absence of a pathogen, cellular byproducts of the damage, termed damage-associated molecular patterns (DAMPs), stimulate pattern recognition receptors (PRRs; Tang et al., 2012). This PRR activation rapidly leads to the activation of resident macrophages, the release of pro-inflammatory cytokines, and ROS production, causing apoptosis of damaged cells and immune cell infiltration (Hume et al., 2001; Park et al., 2004; Tsung et al., 2007). Early inflammation caused by DAMP-PRR signaling is an evolutionarily conserved mechanism for controlling the spread of pathogens or necrotic tissue. In the absence of a pathogen, immune cells are recruited to sites of inflammation to clear debris and facilitate wound healing. Cells of the innate immune system are the first to respond to inflammation. Bone marrow-derived macrophages and neutrophils attempt to kill any damaged cells by nonspecific means, such as by releasing ROS, and they also phagocytose dead and dying cells. This activity mirrors the role of macrophages and neutrophils at the site of an infection, where they kill infected cells to stop the spread of the pathogen. The second wave of cells that infiltrates an area of active inflammation consists of T cells of the adaptive immune system. In the case of inflammation caused by a pathogen, T cells specifically kill cells infected with the pathogen, which they are able to recognize via interaction with a unique T-cell receptor (TCR). T cells also secrete cytokines to modify the activation states of innate cells that are present (Stein et al., 1992). In a sterile inflammatory site, T cells may recognize self-antigens as a result of the cell debris arising from damage and inflammation (Brodeur et al., 2015). T-regulatory cells also infiltrate the site to dampen inflammation and facilitate wound healing (Gazzinelli et al., 1992; Fontenot et al., 2003). In this way, the adaptive immune response first refines then curbs inflammation to bring about an effective resolution. Because inflammatory signaling after noise exposure or ototoxic insult happens quickly in the cochlea and appears to play a role in hair cell death, most research to date has concentrated on preventing the earliest stages of inflammation. However, the immune system is also involved in the resolving inflammation and in wound healing (Nahrendorf et al., 2007; Xu et al., 2014; Lindemans et al., 2015; Psachoulia et al., 2016). It is unclear whether the pro-regenerative resolution of inflammation mediated by the immune response does not occur in the inner ear or whether the cells of the cochlea do not respond to this resolution phase because they are unable to regenerate in response to immune signals.

## Gene Expression in the Ear After Noise Damage

New developments in detecting and sequencing mRNA have enabled the examination of gene transcription after noise damage. This is an important step toward understanding how the cochlea as a whole responds to damage. One of the first studies of transcription after noise damage compared the effect of noise on the lateral wall and organ of Corti in mouse strains that were susceptible or resistant to noise damage (Gratton et al., 2011). An important finding of this study was that C57BL/6 mice, which are susceptible to noise-induced hearing loss, express more genes related to an immune response after noise damage than do mice of resistant strains (Gratton et al., 2011). More recently, a group used RNA sequencing to compare the gene expression in the sensory epithelia of mice and rats 1 day after acoustic injury (Yang et al., 2016). Again, this study highlighted the upregulation of immune-response genes after noise damage, showing that this type of gene expression is conserved across mammalian species. Tan et al. (2016) took this approach a step further by investigating at immune-response gene and protein expression at multiple time points up to 14 days after noise damage. The expression of genes encoding TNF-α, IL-1β and Icam1 increased as early as 6 h after injury with Icam-1 protein remaining elevated at 14 days after noise damage (Tan et al., 2016). Taken together, the results of these studies show that genes encoding cytokines, chemokines and innate immune responses to noise damage are expressed in the cochlea as early as 6 h after damage occurs. Moreover, there was considerable overlap among the genes whose expression was detected in these various studies.

Even though each of the studies described above used different strains of mice, namely CBA/CaJ, 129, C57BL/6, or B6.CAST, it appears that several genes involved in the inflammatory response are expressed following noise damage (Gratton et al., 2011; Tan et al., 2016; Yang et al., 2016). Fos, Socs3, Gpb2 and Icam1 are all associated with the response to noise damage. Socs3 is especially interesting in this regard as it is expressed to dampen JAK/STAT-dependent cytokine signaling by marking signaling components for degradation (Bode et al., 1999; Babon et al., 2008). Regulation of cytokine signaling after damage may control the attraction of new immune cells and the activation of resident immune cells. Icam1 is expressed after NF-κB activation caused by TNF-α and enables recruited immune cells to follow other signals into the cochlea by facilitating the extravasation of the cells from the bloodstream through interaction with lymphocyte function-associated antigen 1 (LFA-1; Wilcox et al., 1990; Wertheimer et al., 1992; Ledebur and Parks, 1995; Suzuki and Harris, 1995). Fos and Gbp2 are both upregulated under conditions of cellular stress and can be expressed in response to interferons (Li et al., 2002; Wei et al., 2008). Although it is important to know that immune response genes can be detected in the cochlear tissue after noise damage, it is much more likely that only a subset of cell types upregulate these genes. Identifying these cell types will enable specific targeting of their contribution to hair cell loss.

## Cell Types that Infiltrate the Cochlea

The first marker used to identify immune cells infiltrating the cochlea after noise damage was CD45, also known as leukocyte common antigen (Kurtin and Pinkus, 1985; Tornabene et al., 2006). Hirose et al. (2005) used the combination of CD45 expression and morphology to characterize most of the infiltrating cells as monocytes or macrophages. Additional markers were subsequently explored, so that today the profile of infiltrating immune cells after noise damage currently includes CD45+, F4/80+, Iba-1+ CD11b+ and CX3CR1+ macrophages (Tornabene et al., 2006; Okano et al., 2008; Tan et al., 2008; Sato et al., 2010; Shi, 2010; Yang et al., 2015). Although these markers are important for identifying macrophage populations, the likelihood of all the infiltrating macrophages and resident macrophages having the same signature is low. The increased sensitivity of cell sorters and flow cytometers will enable a more complete characterization of the expression of these markers on infiltrating cells. The identification of the specific cell types that infiltrate the cochlea is still ongoing, but the timeline of the arrival of these cells after acoustic or ototoxic trauma has been more extensively studied.

**Figure 1:**
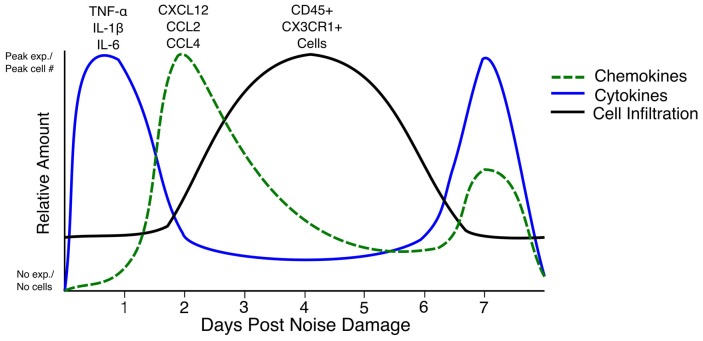
**Timeline of cytokine expression, chemokine expression and cell infiltration in the inner ear after noise damage.** The relative amounts of each cytokine (blue line), chemokine (green dashed line) and cell infiltration (black line) in the first 7 days after noise damage are summarized in the above graph. Note that the cytokine and chemokine expression begins at zero expression as such expression has not been reported in the steady state. The line representing CD45+ and CX3CR1+ cells does not begin at zero as CX3CR1+ and CD45+ cells are present in the cochlea in the steady state. Peak exp.: peak expression. No exp.: No expression.

Few truly comparable studies have examined the combination of cytokine expression, Icam1 expression, and immune cell infiltration with reference to the same parameters of hair cell damage (e.g., hair cell ablation, aminoglycoside/cisplatin ototoxicity and noise damage). This makes it difficult to draw conclusions about the overall immune response after hair cell or cochlear trauma. Nevertheless, it is worth critically synthesizing the information from these disparate studies to inform the future direction of the field. The immune response to each type of damage needs to be characterized, as noise damage affects more cell types than hair cell- specific ablation or aminoglycoside ototoxicity. The best information about the immune response that can be gleaned at present is a rough timeline of events after hair cell death. TNF-α, IL-1β and IL-6 are expressed before as early as 6 h and up to 1 day after damage (Fujioka et al., 2006; Wakabayashi et al., 2010; Tan et al., 2016). Chemokines such as CCL2, CCL4 and CXCL12 are expressed as early as 6 h after damage (Tornabene et al., 2006; Tan et al., 2008, 2016; Dai et al., 2010). Chemokine expression beginning at 24 h after noise seems to be due to ROS, as iNOS-deficient mice do not secrete CXCL12 from the lateral wall after injury to the blood-labyrinth barrier (Dai et al., 2010). By 3–4 days after damage, the numbers of CX3CR1+ and CD45+ cells in the cochlea reach a peak, with increased cell counts being observed until day 7 (Hirose et al., 2005; Kaur et al., 2015). Interestingly, a second peak of expression of TNF-α, IL-1β and IL-6 occurs after cell infiltration, starting at day 3–4 after damage (Oh et al., 2011; Tan et al., 2016). The cytokine expression that occurs as early as 6 h after noise damage could be a result of activation of resident CX3CR1+ macrophages and fibrocytes that are present at the initiation of damage, whereas the secondary peak in cytokine expression could be due to infiltrating immune cells; however, this has not been definitively shown (Figure 1; Okano et al., 2008; Oh et al., 2011; Kaur et al., 2015; Tan et al., 2016). In rats, IL-6 was expressed by type III and type IV fibrocytes of the lateral wall, but not Iba-1 positive macrophages, 6 h after noise damage (Fujioka et al., 2006). Fujioka et al. (2006) also showed that spiral ganglion neurons expressed IL-6 12 h after noise damage. In mice, the receptor for IL-6 and its signal transducer, gp130, are expressed in hair cells in the organ of Corti and the spiral ganglion neurons, meaning that these cells can respond to IL-6 released after noise damage (Wakabayashi et al., 2010). Furthermore, TNF-α, IL-1β and IL-6 staining after lipopolysaccharide (LPS) injection shows expression throughout the cochlea (Oh et al., 2011). The overall effect of these cytokines is to induce the activation of spiral ganglion neurons, lateral wall fibrocytes and immune cells and thereby increase inflammation through the secretion of more of the same cytokines such as TNF-α, IL-1β and IL-6 as well as chemokines such as CCL2 and CXCL12 (So et al., 2007; Dai et al., 2010). However, the specific expression of cytokines and chemokines by immune cells have not been explored. Many of these genes are direct targets of canonical NF-κB, a transcription factor that is upregulated after damage induced by noise or ototoxic drug (Masuda et al., 2005; So et al., 2007). One way in which these cytokines increase immune cell infiltration is by inducing Icam1 expression in the spiral ligament (Tan et al., 2016). Icam1 interacts with receptors on the surface of the immune cell to enable its extravasation into the cochlea (Wilcox et al., 1990; Suzuki and Harris, 1995). Future studies should concentrate on the cell types present and the cytokines expressed immediately after damage, as well as at a 3–4 days and 7–10 days or later after damage, in order to understand the waxing and waning of the whole immune response to damage in the cochlea.

## Evidence for Resident Macrophages

When GFP-labeled bone marrow was used to reconstitute a lethally irradiated mouse, GFP+ bone marrow cells populate the cochlea in the absence of damage to the tissue (Okano et al., 2008). Approximately 80% of these cells were identified as macrophages, based on their morphology and staining for F4/80, Iba-1, CD11b and CD68 (Okano et al., 2008; Sun et al., 2014). Resident macrophages are also positive for CX3CR1, which allows for chemoattraction to CX3CL1 expressed in the organ of Corti and the spiral ganglion; however, the specific chemotaxis of resident macrophages to these sources of CX3CL1 has not been proven (Sato et al., 2008, 2010; Kaur et al., 2015). In the CX3CR1gfp/gfp mouse model, fractalkine signaling is disrupted by GFP knock-in that also labels CX3CR1-expressing cells. Kanamycin treatment in this model results in an increased number of infiltrating CD45+ cells in the cochlea (Sato et al., 2010). Furthermore, transplanting CX3CR1gfp/gfp bone marrow into a wild-type mouse results in a similar phenotype of increased infiltration of CD45+ cells, suggesting that disruption of CX3CR1 signaling on immune cells is detrimental to hair cell survival after aminoglycoside exposure (Sato et al., 2010). When no CX3CR1 is expressed in mice that have a specific loss of all their hair cells, spiral ganglion cell death is increased (Kaur et al., 2015). Taken together, these results indicate that resident CX3CR1+ macrophages may have a valuable role in reducing immune cell infiltration and cell death after aminoglycoside treatment or in response to a specific hair cell lesion. Thus, further characterization of this protective function of CX3CR1+ macrophages will be valuable for understanding the positive role of immune cells in regulating the inflammatory response after cochlear damage.

## TLR4 Activation

Toll-like receptor 4 (TLR4) is a PRR that recognizes multiple ligands, the best characterized of which is Gram-negative bacterial LPS (Liaunardy-Jopeace and Gay, 2014). TLR4 is one of the multiple PRRs that can be activated during sterile inflammation. The downstream effects of TLR4 are ROS production and canonical NF-κB activation (Park et al., 2004; Fan et al., 2007). Several studies have implicated TLR4 activation as one of the pathways leading to inflammation in the cochlea after noise damage or ototoxicity caused by aminoglycoside or cisplatin treatment. Although the exact ligand that activated TLR4 in each of these cases is unknown, each of these types of damage increases the expression of TLR4 in the cochlea within hours (Oh et al., 2011; Hirose et al., 2014; Vethanayagam et al., 2016). In turn, TLR4 activation leads to NF-κB activation and production of TNF-α, IL-1β and IL-6 (Oh et al., 2011). Interestingly, systemic LPS given to mimic a bacterial infection or sepsis amplifies the amount of inflammation in all three types of damage and increases the severity of hearing loss (Oh et al., 2011; Hirose et al., 2014; Vethanayagam et al., 2016). Cochleae deficient in TLR4 exhibit less inflammation, and especially less TNF-α expression, which in turn results in less hearing loss (Oh et al., 2011; Vethanayagam et al., 2016). These studies have furthered our understanding of the negative effects of innate inflammation in the inner ear while raising the possibility that systemic inflammation affects the inflammatory response of the inner ear.

## Immune Modulation

Many mouse models and reagents are available for investigating the modulation of the immune response. Several studies have used these models and techniques in an effort to identify specific cytokines and signaling pathways that are stimulated as a result of cochlear trauma. In the first such study, the FDA-approved anti-TNF-α antibody etanercept was used in conjunction with cisplatin treatment to reduce inflammation in the cochlea (So et al., 2007). Blocking TNF-α release after cisplatin treatment reduced the amount of the canonical NF-κB constituent p65 and the amount of apoptosis throughout the cochlea, even though the outer hair cells were still damaged (So et al., 2007). In a second study, a neutralizing antibody to the IL-6R was used to block the effects of IL-6 released after noise damage (Wakabayashi et al., 2010). This resulted in significantly less infiltration of CD45+ Iba-1+ double-positive cells at day 3 after noise-induced damage, along with less cell death in the spiral ganglion. Furthermore, this study showed that a systemically administered antibody could cross the blood-labyrinth barrier after acoustic trauma, thereby opening the way to studying FDA-approved biologics for treating cochlear damage (Wakabayashi et al., 2010). In a third study, minocycline was used to reduce macrophage activation in order to prevent hair cell damage by CX3CR1+ macrophages (Sun et al., 2014). When minocycline was administered along with neomycin, mice had less microglia-like cell infiltration, less hair cell death, and a reduced threshold shift, which suggests the blockade of macrophage-induced inflammation is important for attenuating the effects of neomycin on hearing (Sun et al., 2014). Although these results appear to indicate that the immune system can be modulated to protect the cochlea from aminoglycoside ototoxicity, follow-up studies in adult mice are needed to verify that this is indeed the case. The most recent study of immune modulation in the cochlea (Vethanayagam et al., 2016) built upon the observation that systemic LPS worsened ototoxic hair cell loss; this study fully examined the role of TLR4 in cochlear inflammation by using TLR4 knockout mice. Compared to wild-type controls, TLR4 knockout mice retained more hair cells and had a lower ABR threshold after noise damage, as well as reduced levels of IL-6 in the organ of Corti (Vethanayagam et al., 2016). Although the TLR4 knockout mice ultimately had less hearing damage, they still exhibited infiltration of macrophages; however, these macrophages did not upregulate MHCII, which would have allowed them to present antigen to the CD4+ T cells of the adaptive immune system (Gloddek et al., 2002; Vethanayagam et al., 2016). Taken together, the results of these studies suggest that the early stimulation of innate receptors and inflammatory cytokines plays a role in hair cell death after damage, although these factors are not solely responsible for all the inflammation that occurs in the cochlea.

## Summary

Thus far, the investigations of the role of the immune system in the inner ear have focused on the early players in inflammation: TLR4 activation, pro-inflammatory cytokine and chemokine release and infiltrating cells of the innate immune system. All three of these major pathways are common to acoustic injury and to ototoxicity caused by both aminoglycosides or cisplatin. This has led researchers in the field to identify ways of systemically modulating the immune system to reduce inflammatory destruction of the inner ear. However, CX3CR1+ resident macrophages appear to regulate the influx of CD45+ cells after hair cell damage. The exact regulatory actions of these cells must be examined to discover ways to dampen damaging inflammation. Also, the role of adaptive immunity in the inner ear is yet to be explored. Infiltrating T cells could prolong inflammation by initiating a self-antigen-specific response (Brodeur et al., 2015). However, in other epithelial systems, adaptive immunity supports tissue regeneration by IL-22 signaling, by dampening the inflammatory response through the release of IL-10, and by polarizing macrophages to an anti-inflammatory phenotype (Gazzinelli et al., 1992; Lindemans et al., 2015; Siqueira Mietto et al., 2015).

Although the mechanisms that act in other tissues may not be applicable to the inner ear, further study is required in three areas in order to learn more about the full extent of the immune response in the inner ear after noise or ototoxic drug damage. First, it is imperative to identify all the cell types that enter the cochlea. Second, once the cell types have been identified, their specific functions must be explored to understand how their secreted products and cell interactions shape the inflammatory response in the inner ear. Finally, each of the PRR families should be investigated to obtain a better understanding of which DAMPs cause the initiation of the inflammatory response in the ear after damage. The results of these studies should reveal new targets for preventative therapies in the case of ototoxic drugs and new treatments for noise-induced hearing loss, and they should expand our fundamental knowledge of the immune response to sterile insults.

## Author Contributions

MBW assembled the literature and wrote the manuscript. JZ saw the need for a review of the material and provided input at every stage of the writing process.

## Funding

This work was supported by the National Institutes of Health (grant numbers 2R01DC006471, 1R01DC015010, R01DC015444, 1R21DC013879 and P30CA21765), ALSAC, the Office of Naval Research (grant numbers N000140911014, N000141210191, N000141210775 and N000141612315) and The Hartwell Foundation (Individual Biomedical Research Award).

## Conflict of Interest Statement

The authors declare that the research was conducted in the absence of any commercial or financial relationships that could be construed as a potential conflict of interest.

## References

[B1] Babon J. J. Sabo J. K. Soetopo A. Yao S. Bailey M. F. Zhang J. G.. (2008). The SOCS box domain of SOCS3: structure and interaction with the elonginBC-cullin5 ubiquitin ligase. J. Mol. Biol. 381, 928–940. 10.1016/j.jmb.2008.06.038 18590740 PMC3652878

[B2] Bánfi B. Malgrange B. Knisz J. Steger K. Dubois-Dauphin M. Krause K. H. (2004). NOX3, a superoxide-generating NADPH oxidase of the inner ear. J. Biol. Chem. 279, 46065–46072. 10.1074/jbc.M403046200 15326186

[B3] Bode J. G. Nimmesgern A. Schmitz J. Schaper F. Schmitt M. Frisch W.. (1999). LPS and TNFα induce SOCS3 mRNA and inhibit IL-6-induced activation of STAT3 in macrophages. FEBS Lett. 463, 365–370. 10.1016/s0014-5793(99)01662-2 10606755

[B4] Brodeur T. Y. Robidoux T. E. Weinstein J. S. Craft J. Swain S. L. Marshak-Rothstein A. (2015). IL-21 promotes pulmonary fibrosis through the induction of pro-fibrotic CD8^+^ T cells. J. Immunol. 195, 5251–5260. 10.4049/jimmunol.1500777 26519529 PMC4655158

[B5] Dai M. Yang Y. Omelchenko I. Nuttall A. L. Kachelmeier A. Xiu R.. (2010). Bone marrow cell recruitment mediated by inducible nitric oxide synthase/stromal cell-derived factor-1α signaling repairs the acoustically damaged cochlear blood-labyrinth barrier. Am. J. Pathol. 177, 3089–3099. 10.2353/ajpath.2010.100340 21057001 PMC2993278

[B6] Fan J. Li Y. Levy R. M. Fan J. J. Hackam D. J. Vodovotz Y.. (2007). Hemorrhagic shock induces NAD(P)H oxidase activation in neutrophils: role of HMGB1-TLR4 signaling. J. Immunol. 178, 6573–6580. 10.4049/jimmunol.178.10.6573 17475888

[B7] Fontenot J. D. Gavin M. A. Rudensky A. Y. (2003). Foxp3 programs the development and function of CD4^+^CD25^+^ regulatory T cells. Nat. Immunol. 4, 330–336. 10.1038/ni904 12612578

[B8] Fredelius L. Rask-Andersen H. (1990). The role of macrophages in the disposal of degeneration products within the organ of Corti after acoustic overstimulation. Acta Otolaryngol. 109, 76–82. 10.3109/00016489009107417 2309562

[B9] Fujioka M. Kanzaki S. Okano H. Masuda M. Ogawa K. Okano H. (2006). Proinflammatory cytokines expression in noise-induced damaged cochlea. J. Neurosci. Res. 83, 575–583. 10.1002/jnr.20764 16429448

[B10] Galea I. Bechmann I. Perry V. H. (2007). What is immune privilege (not)? Trends Immunol. 28, 12–18. 10.1016/j.it.2006.11.004 17129764

[B11] Gazzinelli R. T. Oswald I. P. James S. L. Sher A. (1992). IL-10 inhibits parasite killing and nitrogen oxide production by IFN-gamma-activated macrophages. J. Immunol. 148, 1792–1796. 1541819

[B12] Gloddek B. Bodmer D. Brors D. Keithley E. M. Ryan A. F. (2002). Induction of MHC class II antigens on cells of the inner ear. Audiol. Neurootol. 7, 317–323. 10.1159/000066158 12463193

[B13] Gratton M. Eleftheriadou A. Garcia J. Verduzco E. Martin G. K. Lonsbury-Martin B. L.. (2011). Noise-induced changes in gene expression in the cochleae of mice differing in their susceptibility to noise damage. Hear. Res. 277, 211–226. 10.1016/j.heares.2010.12.014 21187137 PMC3098916

[B14] Hirose K. Discolo C. M. Keasler J. R. Ransohoff R. (2005). Mononuclear phagocytes migrate into the murine cochlea after acoustic trauma. J. Comp. Neurol. 489, 180–194. 10.1002/cne.20619 15983998

[B15] Hirose K. Li S.-Z. Ohlemiller K. K. Ransohoff R. M. (2014). Systemic lipopolysaccharide induces cochlear inflammation and exacerbates the synergistic ototoxicity of kanamycin and furosemide. J. Assoc. Res. Otolaryngol. 15, 555–570. 10.1007/s10162-014-0458-8 24845404 PMC4141430

[B16] Hume D. A. Underhill D. M. Sweet M. J. Ozinsky A. O. Liew F. Y. Aderem A. (2001). Macrophages exposed continuously to lipopolysaccharide and other agonists that act via toll-like receptors exhibit a sustained and additive activation state. BMC Immunol. 2:11. 10.1186/1471-2172-2-11 11686851 PMC58839

[B17] Immig K. Gericke M. Menzel F. Merz F. Krueger M. Schiefenhövel F.. (2015). CD11c-positive cells from brain, spleen, lung and liver exhibit site-specific immune phenotypes and plastically adapt to new environments. Glia 63, 611–625. 10.1002/glia.22771 25471735

[B18] Kaur T. Zamani D. Tong L. Rubel E. W. Ohlemiller K. K. Hirose K.. (2015). Fractalkine signaling regulates macrophage recruitment into the cochlea and promotes the survival of spiral ganglion neurons after selective hair cell lesion. J. Neurosci. 35, 15050–15061. 10.1523/JNEUROSCI.2325-15.2015 26558776 PMC4642237

[B19] Kurtin P. J. Pinkus G. S. (1985). Leukocyte common antigen—a diagnostic discriminant between hematopoietic and nonhematopoietic neoplasms in paraffin sections using monoclonal antibodies: correlation with immunologic studies and ultrastructural localization. Hum. Pathol. 16, 353–365. 10.1016/s0046-8177(85)80229-x 3156803

[B20] Lang H. Nishimoto E. Xing Y. Brown L. N. Noble K. V. Barth J. L.. (2016). Contributions of mouse and human hematopoietic cells to remodeling of the adult auditory nerve after neuron loss. Mol. Ther. 24, 2000–2011. 10.1038/mt.2016.174 27600399 PMC5154482

[B21] Ledebur H. C. Parks T. P. (1995). Transcriptional regulation of the intercellular adhesion molecule-1 gene by inflammatory cytokines in human endothelial cells. Essential roles of a variant NF-κB site and p65 homodimers. J. Biol. Chem. 270, 933–943. 10.1074/jbc.270.2.933 7822333

[B22] Li X. Massa P. E. Hanidu A. Peet G. W. Aro P. Savitt A.. (2002). IKKα, IKKβ and NEMO/IKKγ are each required for the NF-κB-mediated inflammatory response program. J. Biol. Chem. 277, 45129–45140. 10.1074/jbc.M205165200 12221085 PMC1201411

[B23] Liaunardy-Jopeace A. Gay N. J. (2014). Molecular and cellular regulation of toll-like receptor-4 activity induced by lipopolysaccharide ligands. Front. Immunol. 5:473. 10.3389/fimmu.2014.00473 25339952 PMC4186342

[B24] Lindemans C. A. Calafiore M. Mertelsmann A. M. O’Connor M. H. Dudakov J. A. Jenq R. R.. (2015). Interleukin-22 promotes intestinal stem cell-mediated epithelial regeneration. Nature 528, 560–564. 10.1038/nature16460 26649819 PMC4720437

[B25] Ma C. Billings P. Harris J. P. Keithley E. M. (2000). Characterization of an experimentally induced inner ear immune response. Laryngoscope 110, 451–456. 10.1097/00005537-200003000-00024 10718437

[B26] Masuda M. Nagashima R. Kanzaki S. Fujioka M. Ogita K. Ogawa K. (2005). Nuclear factor-kappa B nuclear translocation in the cochlea of mice following acoustic overstimulation. Brain Res. 1068, 237–247. 10.1016/j.brainres.2005.11.020 16376312

[B27] Nahrendorf M. Swirski F. K. Aikawa E. Stangenberg L. Wurdinger T. Figueiredo J. L.. (2007). The healing myocardium sequentially mobilizes two monocyte subsets with divergent and complementary functions. J. Exp. Med. 204, 3037–3047. 10.1084/jem.20070885 18025128 PMC2118517

[B28] Oh G.-S. Kim H.-J. Choi J.-H. Shen A. Kim C.-H. Kim S.-J.. (2011). Activation of lipopolysaccharide-TLR4 signaling accelerates the ototoxic potential of cisplatin in mice. J. Immunol. 186, 1140–1150. 10.4049/jimmunol.1002183 21148032

[B29] Okano T. Nakagawa T. Kita T. Kada S. Yoshimoto M. Nakahata T.. (2008). Bone marrow-derived cells expressing Iba1 are constitutively present as resident tissue macrophages in the mouse cochlea. J. Neurosci. Res. 86, 1758–1767. 10.1002/jnr.21625 18253944

[B30] Park H. S. Jung H. Y. Park E. Y. Kim J. Lee W. J. Bae Y. S. (2004). Cutting edge: direct interaction of TLR4 with NAD(P)H oxidase 4 isozyme is essential for lipopolysaccharide-induced production of reactive oxygen species and activation of NF-κB. J. Immunol. 173, 3589–3593. 10.4049/jimmunol.173.6.3589 15356101

[B31] Psachoulia K. Chamberlain K. A. Heo D. Davis S. E. Paskus J. D. Nanescu S. E.. (2016). IL4I1 augments CNS remyelination and axonal protection by modulating T cell driven inflammation. Brain 139, 3121–3136. 10.1093/brain/aww254 27797811 PMC5382940

[B32] Rock K. L. Latz E. Ontiveros F. Kono H. (2010). The sterile inflammatory response. Annu. Rev. Immunol. 28, 321–342. 10.1146/annurev-immunol-030409-101311 20307211 PMC4315152

[B33] Sato E. Shick E. H. Ransohoff R. M. Hirose K. (2008). Repopulation of cochlear macrophages in murine hematopoietic progenitor cell chimeras: the role of CX3CR1. J. Comp. Neurol. 506, 930–942. 10.1002/cne.21583 18085589

[B34] Sato E. Shick E. H. Ransohoff R. M. Hirose K. (2010). Expression of fractalkine receptor CX3CR1 on cochlear macrophages influences survival of hair cells following ototoxic injury. J. Assoc. Res. Otolaryngol. 11, 223–234. 10.1007/s10162-009-0198-3 19936834 PMC2862920

[B35] Shi X. (2010). Resident macrophages in the cochlear blood-labyrinth barrier and their renewal via migration of bone-marrow-derived cells. Cell Tissue Res. 342, 21–30. 10.1007/s00441-010-1040-2 20838812

[B36] Siqueira Mietto B. Kroner A. Girolami E. I. Santos-Nogueira E. Zhang J. David S. (2015). Role of IL-10 in resolution of inflammation and functional recovery after peripheral nerve injury. J. Neurosci. 35, 16431–16442. 10.1523/JNEUROSCI.2119-15.2015 26674868 PMC6605511

[B37] So H. Kim H. Lee J.-H. Park C. Kim Y. Kim E.. (2007). Cisplatin cytotoxicity of auditory cells requires secretions of proinflammatory cytokines via activation of ERK and NF-κB. J. Assoc. Res. Otolaryngol. 8, 338–355. 10.1007/s10162-007-0084-9 17516123 PMC2538433

[B38] Stein M. Keshav S. Harris N. Gordon S. (1992). Interleukin 4 potently enhances murine macrophage mannose receptor activity: a marker of alternative immunologic macrophage activation. J. Exp. Med. 176, 287–292. 10.1084/jem.176.1.287 1613462 PMC2119288

[B39] Sun S. Yu H. Yu H. Honglin M. Ni W. Zhang Y.. (2014). Inhibition of the activation and recruitment of microglia-like cells protects against neomycin-induced ototoxicity. Mol. Neurobiol. 51, 252–267. 10.1007/s12035-014-8712-y 24781382

[B40] Suzuki M. Harris J. P. (1995). Expression of intercellular adhesion molecule-1 during inner ear inflammation. Ann. Otol. Rhinol. Laryngol. 104, 69–75. 10.1177/000348949510400111 7530436

[B41] Tan B. Lee M. Ruan R. (2008). Bone-marrow-derived cells that home to acoustic deafened cochlea preserved their hematopoietic identity. J. Comp. Neurol. 509, 167–179. 10.1002/cne.21729 18461607

[B42] Tan W. J. T. Thorne P. R. Vlajkovic S. M. (2016). Characterisation of cochlear inflammation in mice following acute and chronic noise exposure. Histochem. Cell Biol. 146, 219–230. 10.1007/s00418-016-1436-5 27109494

[B43] Tang D. Kang R. Coyne C. B. Zeh H. J. Lotze M. T. (2012). PAMPs and DAMPs: signal 0s that spur autophagy and immunity. Immunol. Rev. 249, 158–175. 10.1111/j.1600-065X.2012.01146.x 22889221 PMC3662247

[B44] Taylor A. W. (2016). Ocular immune privilege and transplantation. Front. Immunol. 7:37. 10.3389/fimmu.2016.00037 26904026 PMC4744940

[B45] Tornabene S. V. Sato K. Pham L. Billings P. Keithley E. M. (2006). Immune cell recruitment following acoustic trauma. Hear. Res. 222, 115–124. 10.1016/j.heares.2006.09.004 17081714

[B46] Tsung A. Klune J. R. Zhang X. Jeyabalan G. Cao Z. Peng X.. (2007). HMGB1 release induced by liver ischemia involves Toll-like receptor 4 dependent reactive oxygen species production and calcium-mediated signaling. J. Exp. Med. 204, 2913–2923. 10.1084/jem.20070247 17984303 PMC2118528

[B47] Vethanayagam R. R. Yang W. Dong Y. Hu B. H. (2016). Toll-like receptor 4 modulates the cochlear immune response to acoustic injury. Cell Death Dis. 7:e2245. 10.1038/cddis.2016.156 27253409 PMC5143385

[B48] Wakabayashi K. Fujioka M. Kanzaki S. Okano H. Shibata S. Yamashita D.. (2010). Blockade of interleukin-6 signaling suppressed cochlear inflammatory response and improved hearing impairment in noise-damaged mice cochlea. Neurosci. Res. 66, 345–352. 10.1016/j.neures.2009.12.008 20026135

[B49] Wei L. Fan M. Xu L. Heinrich K. Berry M. W. Homayouni R.. (2008). Bioinformatic analysis reveals cRel as a regulator of a subset of interferon-stimulated genes. J. Interferon Cytokine Res. 28, 541–551. 10.1089/jir.2007.0136 18715197 PMC2988468

[B50] Wertheimer S. J. Myers C. L. Wallace R. W. Parks T. P. (1992). Intercellular adhesion molecule-1 gene expression in human endothelial cells. Differential regulation by tumor necrosis factor-α and phorbol myristate acetate. J. Biol. Chem. 267, 12030–12035. 1351055

[B51] Wilcox C. E. Ward A. M. Evans A. Baker D. Rothlein R. Turk J. L. (1990). Endothelial cell expression of the intercellular adhesion molecule-1 (ICAM-1) in the central nervous system of guinea pigs during acute and chronic relapsing experimental allergic encephalomyelitis. J. Neuroimmunol. 30, 43–51. 10.1016/0165-5728(90)90051-n 1977768

[B52] Xu M. J. Feng D. Wang H. Guan Y. Yan X. Gao B. (2014). IL-22 ameliorates renal ischemia-reperfusion injury by targeting proximal tubule epithelium. J. Am. Soc. Nephrol. 25, 967–977. 10.1681/ASN.2013060611 24459233 PMC4005304

[B53] Yang S. Cai Q. Vethanayagam R. R. Wang J. Yang W. Hu B. H. (2016). Immune defense is the primary function associated with the differentially expressed genes in the cochlea following acoustic trauma. Hear. Res. 333, 283–294. 10.1016/j.heares.2015.10.010 26520584 PMC4798880

[B54] Yang W. Vethanayagam R. R. Dong Y. Cai Q. Hu B. H. (2015). Activation of the antigen presentation function of mononuclear phagocyte populations associated with the basilar membrane of the cochlea after acoustic overstimulation. Neuroscience 303, 1–15. 10.1016/j.neuroscience.2015.05.081 26102003 PMC4532582

